# The search for *cis*-regulatory driver mutations in cancer genomes

**DOI:** 10.18632/oncotarget.5085

**Published:** 2015-08-20

**Authors:** Rebecca C. Poulos, Mathew A. Sloane, Luke B. Hesson, Jason W. H. Wong

**Affiliations:** ^1^ Prince of Wales Clinical School and Lowy Cancer Research Centre, UNSW Australia, Sydney, Australia

**Keywords:** promoter, enhancer, cancer, somatic mutation, *cis*-regulation

## Abstract

With the advent of high-throughput and relatively inexpensive whole-genome sequencing technology, the focus of cancer research has begun to shift toward analyses of somatic mutations in non-coding *cis*-regulatory elements of the cancer genome. *Cis*-regulatory elements play an important role in gene regulation, with mutations in these elements potentially resulting in changes to the expression of linked genes. The recent discoveries of recurrent *TERT* promoter mutations in melanoma, and recurrent mutations that create a super-enhancer regulating *TAL1* expression in T-cell acute lymphoblastic leukaemia (T-ALL), have sparked significant interest in the search for other somatic *cis*-regulatory mutations driving cancer development. In this review, we look more closely at the *TERT* promoter and *TAL1* enhancer alterations and use these examples to ask whether other *cis*-regulatory mutations may play a role in cancer susceptibility. In doing so, we make observations from the data emerging from recent research in this field, and describe the experimental and analytical approaches which could be adopted in the hope of better uncovering the true functional significance of somatic *cis*-regulatory mutations in cancer.

## INTRODUCTION

The field of cancer research has expanded remarkably since it was first suggested over a century ago, that cancer is caused by chromosomal abnormalities [1]. In the last few decades, numerous driver mutations have been identified, and comprehensive lists of cancer-associated genes have been developed [2]. The primary research focus until recently has been almost entirely upon somatic mutations that lie within coding regions, which account for only ∼2% of the genome. In the last few years however, there has been significant interest in somatic cancer mutations arising in the remaining 98% of the human genome which is non-coding. This expansion in focus has been driven primarily by advances in sequencing and other genomic technologies which have allowed scientists to mine previously unexplored regions of the genome. For example, the costs of sequencing a whole human genome have dropped rapidly in the past decade, with some sequencing endeavours having finally reached the famed US$1,000 mark [3]. These technological advances and reductions in sequencing costs mean that it is no longer a technical or financial barrier to sequence the entire genome of a large number of human cancers, and perform large-scale analyses with statistically significant outcomes.

In addition to reduced sequencing costs, technologies have advanced to allow for the increasingly accurate and detailed identification of regulatory regions in the non-coding genome. This is particularly important since the Encyclopedia of DNA Elements (ENCODE) project estimates that as much as 80% of the human genome may be functional [4], highlighting the potential relevance of somatic mutations within the non-coding genome. A comprehensive recent review of current computational methods available to identify *cis*-regulatory regions in the genome can be found at [5], and thus will not be addressed in this review.

Despite these significant advances, substantial challenges still remain in the interpretation of the findings of such non-coding genome-sequencing endeavours. For example, whole-genome sequencing (WGS) has revealed that generally, intergenic DNA shows a rate of mutation which is almost twice as high as the rate in coding DNA, possibly due to a lack of selective pressure in non-coding regions [6], but also due to different rates and mechanisms of DNA repair across the genome [7–10]. This higher mutation rate makes it particularly difficult for researchers to identify driver non-coding mutations amongst the vast background of passenger mutations [2]. Furthermore, determining how a given mutation-harbouring region regulates expression, and which genes are affected, remains a major challenge.

In this review, we focus on the recent developments that have been made in the attempt to identify driver somatic mutations in *cis*-regulatory regions of the cancer genome. We first describe the initial discoveries of somatic *cis*-regulatory mutations occurring in sporadic cancers. We next make observations from the data that has emerged from recent discoveries, and critically review the methodology used to identify these non-coding mutations. Finally, we propose recommendations for future studies aimed at identifying and validating functionally relevant *cis*-regulatory mutations in the context of cancer.

## FEATURES OF *CIS*-REGULATORY REGIONS

DNA in the eukaryotic genome is organised into chains of nucleosomes called chromatin [11]. Each nucleosome consists of approximately 147 bp of DNA wrapped around an octamer of histone proteins [12]. Nucleosomes package around two metres of DNA into the nucleus of each cell, with their precise positioning playing an important role in the regulation of DNA function, including DNA replication, repair and the expression of genes [13].

**Figure 1:**
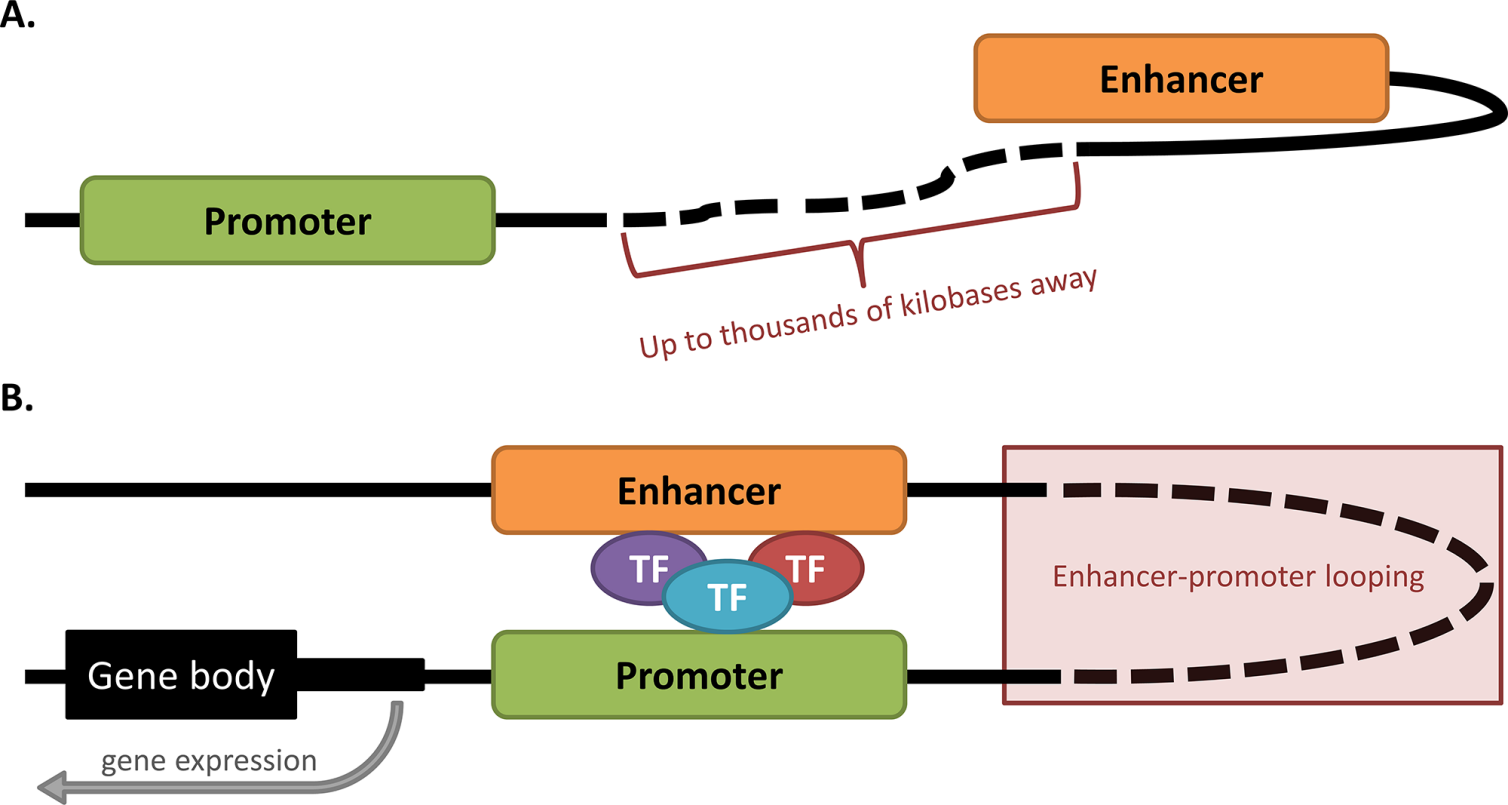
Enhancer-promoter looping occurs over vast distances of DNA **A.** Inactive enhancer and promoter. The enhancer for a given gene can potentially lie up to thousands of kilobases away from the promoter which it regulates. When in the inactive state, the promoter and enhancer may not be in close proximity. **B.** Enhancer-promoter looping. In the active state, the enhancer and promoter form a loop of DNA, enabling the enhancer to contact the promoter and recruit transcription factors (TFs) to the region, leading to gene expression. The DNA loop can form even when the enhancer and promoter are located at vast distances from each other.

Gene promoters and enhancers are examples of *cis*-regulatory regions (Table 1) that often show nucleosome depletion. At the promoters of highly expressed genes, nucleosomes are located just upstream and downstream of the transcription start site (TSS), thereby creating a nucleosome depleted region. This feature is thought to facilitate access for transcription factors (TFs) to bind [14]. TFs are *trans*-acting DNA binding proteins that bind regulatory elements, either enhancing or repressing transcription [15]. The function of a gene promoter can be influenced by other regulatory regions such as enhancers which, when active, also show nucleosome depletion and can recruit TFs. Enhancers can physically interact with gene promoters by looping the intervening chromatin to juxtapose the two regions (Figure 1). This allows enhancers to regulate the activity of promoters that may be several hundred kilobases away. One hypothesis is that variants or somatic mutations occurring in *cis*-regulatory regions can impact upon gene expression by altering the unique binding sites that are recognised by TFs.

**Table 1:** Types of *cis*-regulatory elements and their definitions

*Cis*-regulatory element	Definition
Promoter	The core promoter is the DNA region to which transcriptional machinery binds [15]. It includes some of the following: TATA box, initiator element (Inr), and downstream promoter element [15]. The Inr is the most common feature, being present in approximately half of all promoters [91]. Proximal promoter elements typically lie immediately upstream and within close proximity of the core promoter element [15].
Enhancer	Enhancers are specific DNA sequences that can regulate the activity of a promoter. The first enhancer was discovered in the SV40 tumour virus genome [92], and the first human enhancer was identified in the immunoglobulin heavy-chain locus [93]. Secondary enhancers have also been identified, and may act as ‘shadow enhancers’, ensuring that enhancer activity continues even if environmental conditions change and affect primary enhancer function [94–96].
Super-enhancer	Super-enhancers are defined as a grouping of enhancers that are situated within close proximity of each other and combinatorially bind transcription factors [76]. They tend to be differentiated from regular enhancers through a particular occupancy by cofactors [76]. However, there is still debate about whether super-enhancers really are a truly separate class of regulatory region, or whether they are simply strong enhancers that operate generally in the same way as a typical enhancer [97].
Insulator	Insulators are DNA sequences that act to partition the genome into regions defined by transcriptional activity [15].
Silencer	Silencers are specific DNA sequences that halt transcription by serving as binding sites for negative transcription factors (also called ‘repressors’) [15].

Nucleosome depleted regions can be detected as DNase I hypersensitive (DHS) sites due to their sensitivity to cleavage by the DNase I enzyme. DHS regions can be identified genome-wide by DNase I sequencing (DNase-seq). Enhancers and promoters can be differentiated through the signature of specific histone marks flanking the DHS, which can be identified by chromatin immunoprecipitation sequencing (ChIP-seq). For example, H3K4me3 and H3K4me1 typically mark promoter and enhancer regions respectively [16, 17]. In addition, H3K27ac and H3K9me3 identify activated and repressed *cis*-regulatory regions respectively [16–18]. Therefore, the combinatorial use of DNase-seq and ChIP-seq allows researchers to identify the nucleosome occupancy and specific histone marks which define the presence and activity of certain *cis*-regulatory regions. In addition, ChIP-exo can be adopted. This technique was recently used to locate somatic cancer mutations in TF binding sites [19] as it can identify, at almost base pair resolution, the binding locations of DNA-binding proteins [20]. Techniques such as these can help to accurately identify a *cis*-regulatory region within which a mutation lies, and so allow for a better determination of the analyses which may be needed to assess the functional role of such mutations within the cancer genome.

## INITIAL DISCOVERIES: SOMATIC MUTATIONS IN *CIS*-REGULATORY REGIONS IN CANCER

The primary focus of this review is on the role of somatic point mutations and small insertions or deletions (indels) within regulatory regions in cancer. We do not focus on the role of large-scale structural rearrangements (due to the differences in the techniques needed to identify and analyse these forms of variation), nor do we address germline variation in detail. Though, it is worth highlighting that most single nucleotide polymorphisms (SNPs) identified by genome-wide association studies (GWAS) to be significantly associated with cancer and disease are located within non-coding regions of the genome [21]. In contrast to specific somatically acquired mutations however, germline variants may be part of a larger haplotype in linkage disequilibrium. Therefore, it is not always possible to pinpoint the pathogenic germline variant amongst several within a haplotype. Even so, multiple examples exist of SNPs in putative *cis*-regulatory regions which are linked with increased risk of cancer development, for examples see [22–34]. This suggests that we should also expect to find somatic *cis*-regulatory mutations that play a role in cancer development. Despite this, the prevalence of somatic *cis*-regulatory mutations as cancer drivers has not yet been established. In this section, we discuss the initial discoveries of such mutations as they are relevant to further research conducted in the field.

### *TERT* promoter mutations acting as cancer drivers

**Figure 2:**
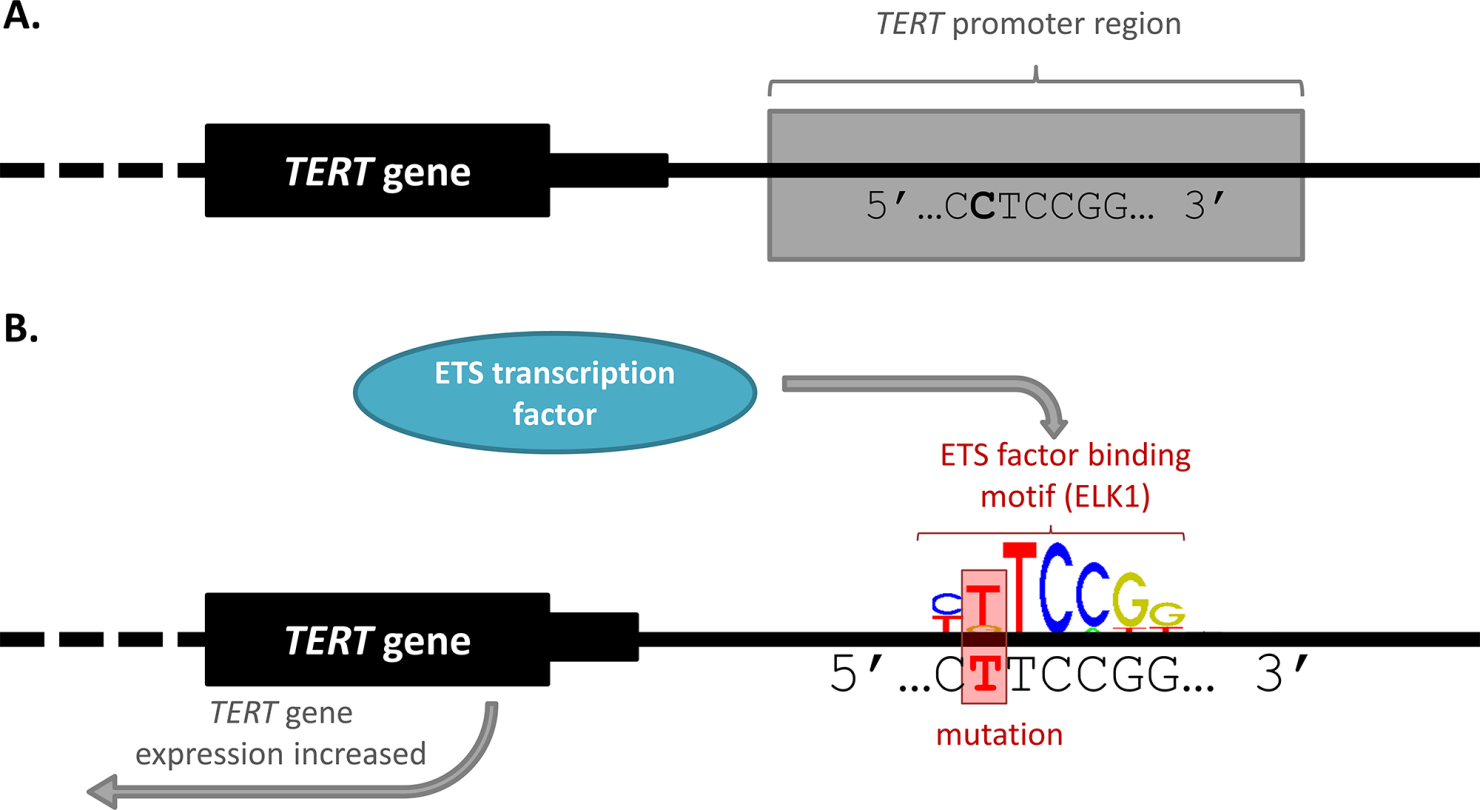
*TERT* promoter mutation alters transcription factor binding and gene expression **A.** Wild-type *TERT* promoter. The *TERT* gene body is marked by a black box, with the intronic region identified by a dotted line. The wild-type DNA sequence for a small portion of the promoter region is indicated. **B.** Mutant *TERT* promoter. The mutated *TERT* promoter sequence is given, featuring a C > T mutation which creates a consensus binding motif for an ETS transcription factor. The sequence created is identical for both the chr5:1,295,228 and chr5:1,295,250 C > T mutations identified by Huang, *et al.* [35]. The first 7 bases of the ELK1 (ETS family) binding motif is shown for illustrative purposes (obtained from the Jaspar database [88]). This image indicates the way in which the mutations can create a binding site for an ETS transcription factor, leading to transcription factor binding and increased *TERT* gene expression.

The first and arguably most significant discovery of somatic *cis*-regulatory mutations in cancer were recurrent somatic mutations found in the promoter of the telomerase reverse transcriptase *(TERT)* gene. In 2013, two articles [23, 35] were published simultaneously that documented independent discoveries of cancer-associated variation within the *TERT* promoter. Two recurrent somatic mutations (chr5:1,295,228 C > T and chr5:1,295,250 C > T) were identified in 71% of WGS malignant melanomas analysed [35]. The *TERT* promoter mutations were initially identified as worthy of further research because they were highly recurrent, mutually exclusive with each other, and occurred in the absence of a high background of passenger mutations in the surrounding region [35]. The mutations alter the expression of *TERT* by creating *de novo* motifs for the binding of GA-binding protein (GABP) which is part of the E twenty-six (ETS) family of TFs [35, 36] (Figure 2). A germline mutation (chr5:1,295,161 T > G) was also found in the *TERT* promoter which segregated disease in individuals in a melanoma-prone family [23].

It is well established that cancer cells have high telomerase activity levels, but few coding mutations have been identified within the *TERT* gene [37]. However, over-expression of *TERT* enables telomere renewal, which is necessary for cellular immortalisation, a hallmark of cancer [38]. This seminal finding represented the first identification of recurrent somatic mutations within a promoter region in cancer [35] and has led to further studies aimed at determining the prevalence of *TERT* promoter mutations in other cancers [39–44]. In the past two years, the same two somatic *TERT* promoter mutations, together with additional *TERT* promoter mutations, have been identified in numerous other cancers, with particularly high prevalence in glioblastoma (62%) and bladder cancer (59%) [45]. The clinical significance of these findings is highlighted by the current investigation of *TERT* promoter mutations as potential biomarkers for cancer prognosis [37].

### Enhancer-altering mutations in the development of leukaemia

In 2014, small heterozygous somatic insertions containing TF motifs for the MYB transcription factor were identified in tissue samples and cell lines of T-cell acute lymphoblastic leukaemia (T-ALL) [46]. The mutations cause the spontaneous formation of a super-enhancer capable of binding MYB, recruiting other important TFs and causing mono-allelic overexpression of T-cell acute lymphocytic leukemic protein 1 *(TAL1)* [46]. These mutations thus drive tumorigenesis in T-ALL, having been discovered in an attempt to account for the mono-allelic overexpression which had been observed in some T-ALL samples despite a lack of any translocations at the *TAL1* locus or TAL1 abnormalities [46, 47]. This research is highly significant as it is the first description of somatic driver mutations which affect enhancers in cancer [48] and thus uncovered a mechanism in carcinogenesis which is potentially common but yet to be characterized [46].

While structural variation is not the focus of this review, it is still worth noting the recent identification of recurrent 3q rearrangements in some acute myeloid leukaemia (AML) samples [49]. These rearrangements result in the repositioning of an enhancer element which causes cancer development by simultaneously activating *EVI1* and causing haplo-insufficiency of *GATA2* in AML [49]. As chromosomal rearrangements are a factor in virtually all cancer types [50], this finding suggests that the structural rearrangement of enhancer elements may be a potentially common mechanism of cancer development.

## OBSERVATIONS FROM RECENT DISCOVERIES

The identification of the recurrent *TERT* and *TAL1* mutations raises the possibility that other *cis*-regulatory regions may acquire somatic mutations that contribute to cancer by similar mechanisms. By understanding the methodology adopted in the discovery of these mutations, researchers are better able to investigate whether other *cis*-regulatory mutations are functionally relevant in cancer development. However, despite scientifically rigorous analyses of WGS data from a range of cancers [6, 51, 52], non-coding somatic mutations have yet to be identified with such robust links to cancer development as the *TERT* and *TAL1* regulatory examples. In the following sections, we describe the research efforts undertaken to identify further examples of *cis*-regulatory somatic mutations in cancer. We conclude by drawing together these findings to make recommendations for future research directions.

### High numbers of somatic point mutations in *cis*-regulatory regions of cancer genomes

It has been established that the somatic cancer mutation rate in DHS regions is generally lower than in other genomic regions due to increased accessibility of regulatory DNA by repair mechanisms [9]. Nevertheless, recent research has indicated that there are a large number of somatic cancer mutations in regulatory DNA. For example, Melton *et al*. [52] found that after correcting for mapping errors, across cancer types, almost 40% of somatic mutations were within portions of the genome annotated to be regulatory. Further, Mathelier *et al*. [53] found enrichment for somatic point mutations within TF binding sites when compared with coding exons in a majority of B-cell lymphoma samples. While most regulatory mutations are likely to be passengers, the shear prevalence of somatic mutations in regulatory regions of cancer genomes, together with the large portion of the genome that may have regulatory function, highlights the important pool of candidate mutations from which cancer drivers may yet be identified. Interestingly, it is worth noting that the same pattern of elevated rates of somatic point mutations in regulatory regions has not been observed with respect to indels [53], suggesting that a different mechanism that may be at play regarding indel accumulation, repair or selection.

Despite the high number of somatic point mutations in regulatory regions, the impact of such mutations on gene expression is yet to be determined genome-wide. However, recent attempts have been made to establish the portion of regulatory mutations which are functional in a single genome [54]. Poulos *et al*. [54] used reporter assays to screen promoter mutations in an unbiased manner within the melanoma cell line COLO-829, finding that almost 20% of mutations altered promoter activity in mutant compared to wild-type sequences. The high number of somatic cancer mutations in regulatory regions, together with the relatively high percentage of functional promoter mutations identified in a single genome, highlights the urgent need to consider the role that these mutations may have on cancer development.

### Promoter mutations in other cancer-associated genes

Many potentially important *cis*-regulatory mutations have been identified proximally to cancer-associated genes. These mutations were prioritised since they lie in mutational hotspots [6], are recurrent [51] or are linked with an expression change in an associated gene [55]. Further establishing this association, in a cohort of B-cell lymphoma samples, Mathelier *et al*. [53] found that genes harbouring mutations in TF binding sites within their promoters were significantly enriched for genes in apoptosis and other oncogenic pathways. The high prevalence of mutations in the regulatory regions of genes involved in cancer-associated pathways has been highlighted in the published literature on numerous occasions [6, 52, 53, 55, 56]. Some of these analyses simply noted that cancer-related genes were included within their findings, while others performed some form of statistical measure. However, it would be of great interest to determine whether somatic mutations in regulatory regions of cancer-related genes are truly enriched in cancer genomes across multiple cancer types, and whether this is particularly the case in either promoters or enhancers.

One explanation for the identification of many promoter mutations in cancer-associated genes is that these genes may be more sensitive to the effects of point mutations in their promoters. Perhaps such mutations result in small but important changes in expression of these potential onco- or tumour-suppressor-genes. As a result, the mutations may be selected for within proliferating cancer cells and so drive cancer development. However, the distinct mutagenic potential of the *cis*-regulatory regions of cancer-associated genes is yet to be determined.

### Recurrent mutations are often not associated with gene expression change

While a number of genes have been found to harbour recurrent mutations, many, including cancer-associated genes, do not have clear links with expression change [6, 51, 52]. Even in cases where increased somatic mutation accumulation in a regulatory region has been correlated with a change in expression of the associated gene, causation has been difficult to establish [56]. As an example of this issue, Fredriksson *et al*. [51] used WGS data from 14 different cancers to identify mutations within 500 bp of a TSS that were recurrent in at least 5 tumours, but which existed in a ± 5 kb region of low overall mutation density. Two genes found to have nearby mutations are *PLEKHS1* and *DPH3. PLEKHS1* harbours two mutations close to its TSS (chr10:115,511,590 and chr10:115,511,593, both predominantly C > T [6]), while the region surrounding the *DPH3*-proximal mutations (most recurrent at chr3:16,306,505 C > T) had the lowest background mutation rate of all the recurrent mutations identified [51]. Despite strong selection criteria being applied, and the recurrence of the mutations identified, *PLEKHS1* was not over- or under- expressed in mutant samples when compared to wild-type [51]. Regarding *DPH3*, the gene appeared to be more highly expressed in *DPH3* promoter mutants than wild-type samples in a small cohort (*n* = 38) of melanomas, but no difference in expression was observed when a larger cohort (*n* = 173) was interrogated [51]. This finding is unexpected, particularly considering the similarities that exist between the *TERT* promoter mutations and the *DPH3-*proximal mutations: *DPH3* is a candidate cancer-associated gene (a potential tumour-suppressor); the mutations are recurrent in melanomas (13%); and the mutations alter a predicted ETS TF binding site [51]. With such features, it would otherwise be expected that the *DPH3*-proximal mutations would be prime candidates to be a potential driver in cancer development. However, the lack of association with expression changes suggests that a more complex interaction may be at play.

Fredriksson *et al*. [51] suggested a number of alternate hypotheses to account for the lack of correlation between the recurrent *DPH3*-proximal mutations and expression. These hypotheses should also be considered when assessing other *cis-*regulatory mutations. For example, temporal patterns in expression may alter during cell-cycle progression or under specific conditions, such as at times of cellular stress. As such, a mutation may only become relevant under certain conditions. Alternatively, a mutation may co-operate with other mutations in the genome, in a similar way to SNP interactions in germline DNA. In fact, both the *DPH3*-proximal mutations and the *TERT* promoter mutations significantly co-occur with *NF1* and *BRAF* mutations, respectively [51]. Compound or cell-cycle-specific gene expression effects from somatic cancer driver mutations in *cis*-regulatory regions are yet to be established. However, if these hypotheses are correct, the effects of any mutation on gene expression could be subtle and not readily measured in heterogeneous tissues. Importantly, this would limit the effectiveness of non-specific genome-wide gene expression analysis – a common tool used today from databases such as The Cancer Genome Atlas (TCGA) or the International Cancer Genome Consortium (ICGC) [57]. These suggestions are supported by the results of a survey of somatic promoter mutations in the genome of the melanoma cell line COLO-829 [54]. This study found a mutation in the promoter of *NDUFB9* which was responsible for decreased promoter activity in reporter assays [54]. The mutation was recurrent in other melanoma genomes and significantly co-occurred with coding *NF1* mutations, but a corresponding association with altered gene expression was not apparent in mutant samples from a TCGA cohort [54].

In addition to biological explanations, samples size and statistical power remain limiting factors when performing large-scale analyses of regulatory mutations in cancer [6, 51, 52]. When mutations are present at low frequency or in only a single cancer type, the mutant sample sizes available for expression analyses make robust conclusions difficult to reach. This is further compounded by the need to perform extensive corrections for multiple testing when determining genome-wide expression correlations, requiring that the strength of associations between mutation and expression change be highly significant. This issue may be partially overcome as sample sizes increase and new statistical methods are developed to analyse such data.

An alternative interpretation of the data however, is that recurrence alone may not be a good discriminator between functional and silent mutations in the non-coding genome. Instead, mutation recurrence within the non-coding genome may more often implicate bases that are particularly prone to mutagenesis than it does cancer driver mutations. This could particularly be the case in *cis*-regulatory regions, as TF binding may induce mutagenesis and prevent DNA repair [58, 59], and could account for mutations which are recurrent in the absence of functional consequence. The interaction between TF binding, DNA repair and somatic mutation accumulation is yet to be fully elucidated, but this consideration highlights the need to functionally validate the role in cancer development of any recurrent mutations identified.

## RECOMMENDED STRATEGIES FOR IDENTIFYING FUNCTIONAL *CIS*-REGULATORY MUTATIONS

### The need to develop a research pipeline with appropriate experimental validation

Research into the functional impact of somatic *cis*-regulatory mutations in cancer is still in its infancy, with only a handful of studies performed to date, for examples see [6, 51–54, 56, 60]. However, it has already become apparent that when different criteria are applied to prioritise candidate mutations for further analysis from a somewhat common pool, different sets of mutations can be highlighted [6, 52]. This can be partly attributed to differences in the samples used for analyses, with many WGS sample sizes from a single cancer type still being less than ideal. However, in many cases, different window sizes have been used in order to determine regulatory regions that are recurrently mutated. For example, some criteria that have been used to determine regional recurrence include mutations within 50 bp of each other [6] or mutations within windows of 10 bp [52], 100 bp [51] or up to 1 kb [53]. Additionally, Smith *et al*. [56] developed a computational method known as SASE-hunter to study signatures of accelerated somatic evolution (SASE). This method was particularly applied to study SASE within a 6 kb region (−5 kb to +1 kb of the TSS) to identify promoter regions with more mutations than expected by chance alone [56]. Consideration of regional recurrence in addition to base-pair recurrence is highly important, but this variation in the size of the regions focused upon in analysis demonstrates the need for the development and validation of a research pipeline which can accurately identify biologically relevant clusters of cancer driver mutations from among the vast background of passenger mutations in the non-coding cancer genome.

**Figure 3:**
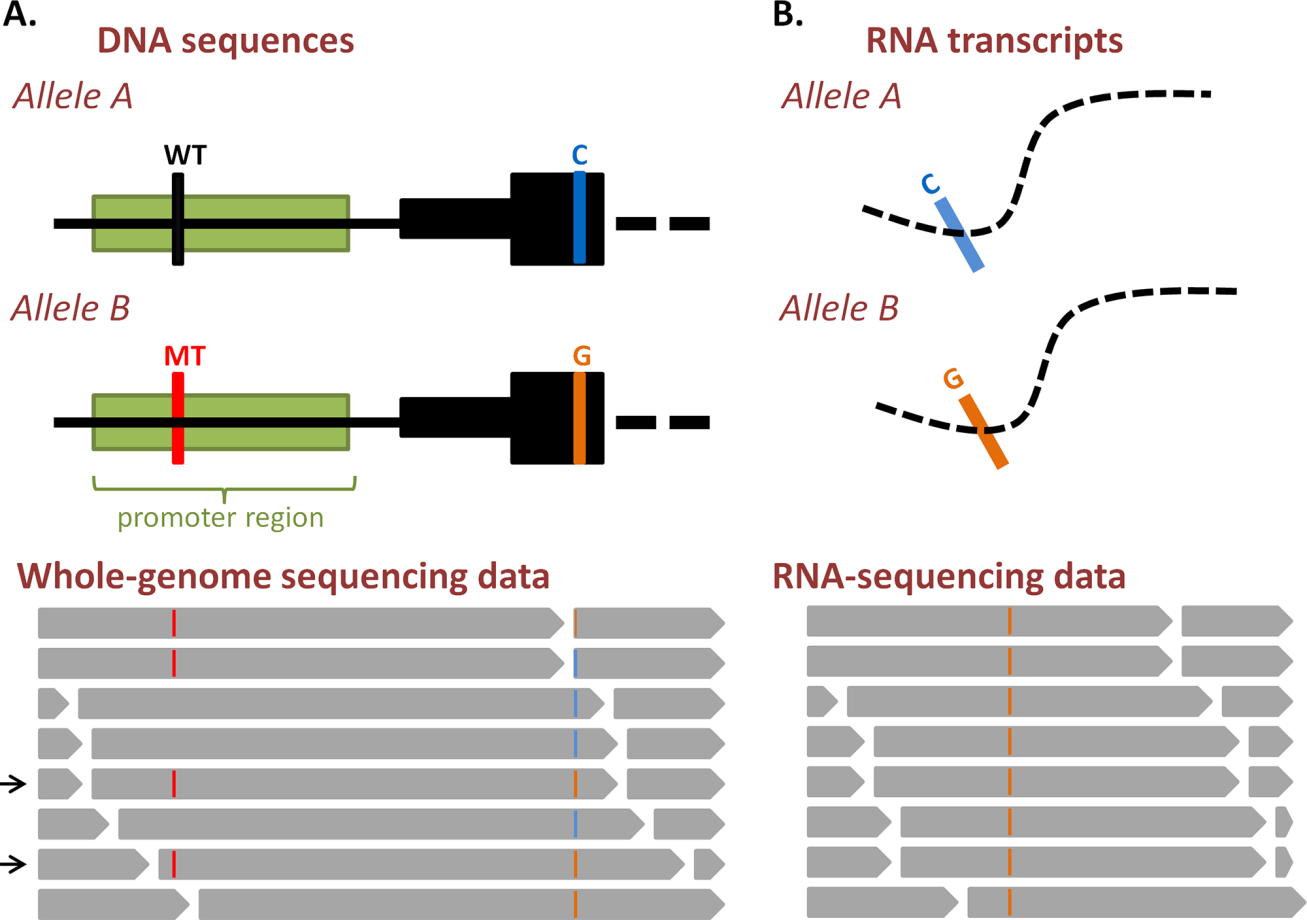
Analysis of allele-specific gene expression from DNA- and RNA-sequencing data **A.** DNA and whole-genome sequencing data. An example of a promoter mutation (red vertical bar) identified by whole-genome sequencing. This mutation is heterozygous and is *in cis* (i.e. on the same allele: allele B) with an informative SNP (vertical orange bar) within exon 1 of the gene. This is determined by the presence of both variants on single DNA molecules (indicated by arrows) from the whole-genome sequencing reads (grey bars, lower panel). **B.** RNA and RNA-sequencing data. Figure shows detection of the same informative SNP in transcribed molecules using RNA-sequencing. The relative expression of the two alleles is determined by comparing the number of sequenced molecules containing this variable SNP (grey bars, lower panel). This in turn infers the potential impact of the promoter mutation on gene expression. In this case, the promoter mutation appears to be associated with activation of the expression of this allele.

Such a research pipeline will undoubtedly involve experimental validation in a relevant *in vivo* biological context to distinguish between functional and silent *cis*-regulatory somatic mutations. This experimental validation could take on a variety of forms. For example, both of the landmark studies of *TERT* promoter mutations in melanoma [23, 35] used luciferase reporter assays to show increased activity of mutant over wild-type sequences. Additionally, the *TAL1* [46] and *GATA2/EVI1* [49] enhancer studies both utilised CRISPR/Cas genome editing technology [61] to show that the enhancer alterations were causative of the gene expression changes they identified. Notably also, allele-specific expression analyses utilising RNA-seq (Figure 3) or pyrosequencing [62] can be further used to demonstrate the pathogenic role of a somatic *cis*-regulatory mutation *in vivo*. This type of analysis can take on a variety of forms, with RNA-seq data having been used recently in a study of colorectal cancers [63]. This study investigated the allelic ratios between samples in heterozygous locations, in order to identify genes with somatic alterations in their regulatory regions [63].

More recent work in the field has utilised genome-wide RNA-seq data from TCGA database rather than site-specific experimental validation to determine gene expression changes. Experimental analysis in the form of reporter assays has been used in a limited number of circumstances [52, 54, 55], but many studies have taken purely bioinformatic approaches. While these computational analyses were scientifically rigorous and TCGA transcriptomic data is a useful resource, as discussed previously, it is yet to be established whether analyses of heterogeneous whole-tumour data alone are sufficient to uncover truly functional *cis*-regulatory somatic mutations with potentially subtle impacts on gene expression, in the absence of targeted experimental work. This fact is particularly notable with regard to the recurrent mutations proximal to *PLEKHS1* that were discussed previously. These mutations were separately identified in two independent studies [6, 51] but, despite both using TCGA bladder cancer gene expression data, Fredriksson *et al*. [51] concluded no change in expression between wild-type and mutant groups, while Weinhold *et al*. [6] found a significant decrease in expression in mutant samples when compared to wild-type. It remains to be seen whether any expression change (or lack thereof) will persist in experimental scenarios for the *PLEKHS1*-proximal mutations. A validated research pipeline, once developed, will indicate the most appropriate type of experimental analysis that ought to accompany bioinformatic predictions of functional *cis*-regulatory mutations.

### Transcription factor motif alteration and gene expression changes

The *TERT* promoter mutations create motifs for ETS TF binding, and so it was hypothesised that increased binding of ETS factors in the mutated promoter led to increased *TERT* expression and cellular immortalization [35] (Figure 2). Subsequent studies have adopted slightly different methodologies and criteria in their research, but two studies used gene expression data to specifically investigate somatic mutations that alter ETS factor motifs [6, 51]. This method of analysis interestingly led to the identification of a potential link between the *TERT* promoter and control of *CLPTM1L* gene expression [51]. A systematic analysis of the mutations that alter other important TF motifs, in addition to ETS, may identify further promising candidate cancer driver mutations and associations worthy of investigation [6]. In fact, subsequent analyses of mutations in other TF motifs has led to the finding that CTCF/cohesion [19] and CEBP [52] binding sites are significantly mutated – both with potential links to tumorigenesis. Interestingly, such analyses of other TF motifs, has also noted that some specific bases in a given motif are more often mutated, suggesting a potential underlying selective pressure on such mutations [52].

**Figure 4:**
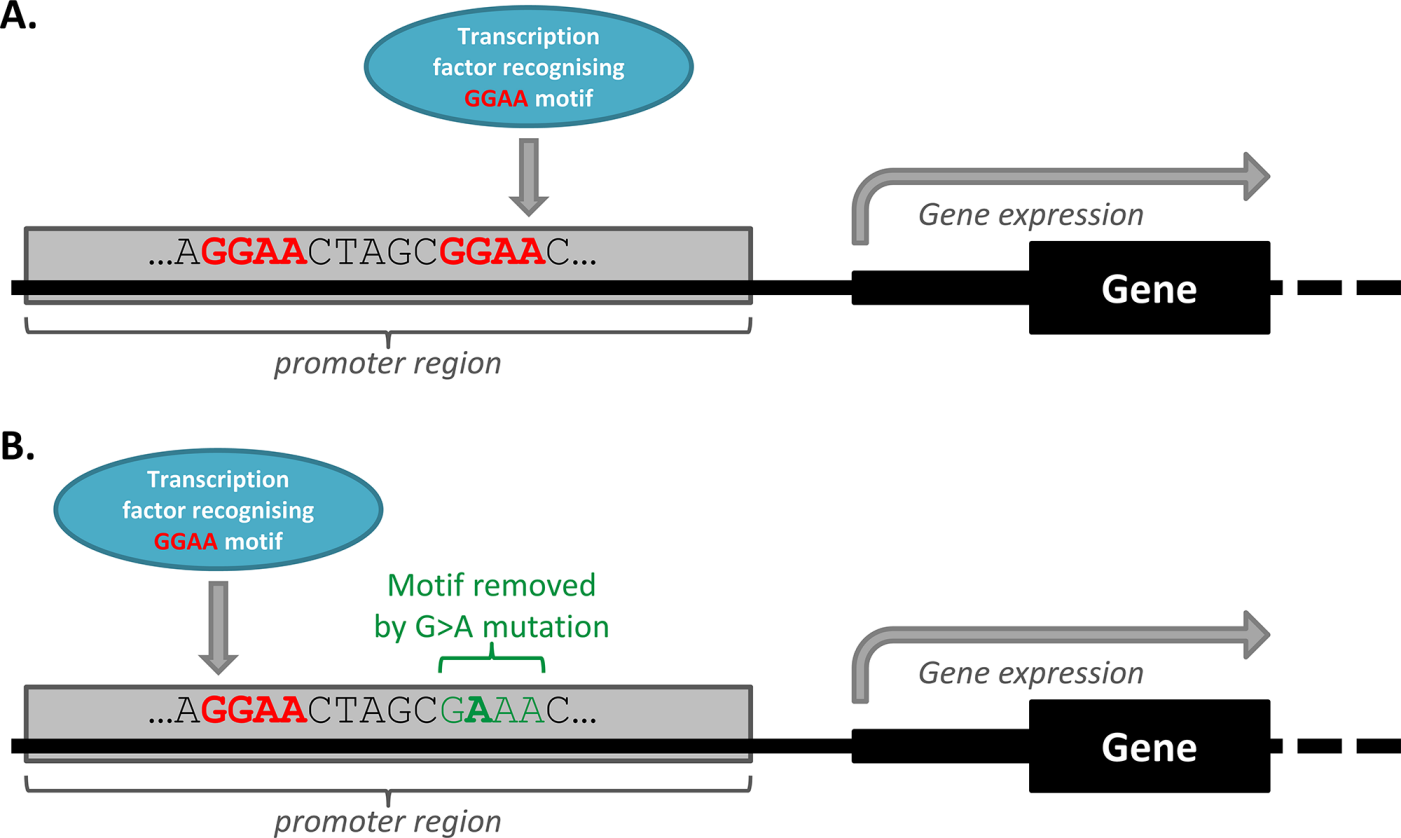
Transcription factor redundancy can impact on the functional effect of some mutations **A.** Wild-type promoter, with transcription factor redundancy. The wild-type promoter region depicted in this image has two GGAA motifs which can be recognised by the illustrated transcription factor. Only one motif is being utilised by the wild-type promoter, and the gene is being expressed. **B.** Mutant promoter, with transcription factor redundancy allowing for compensatory binding. A G > A somatic mutation means that the mutant promoter region has lost one GGAA motif. However, in this simplified scenario, the illustrated transcription factor will bind instead to the GGAA motif that was previously redundant in the wild-type promoter, meaning that gene expression does not change in the associated gene.

In all analyses of mutations in TF motifs however, TF motif redundancy ought to be considered. TF motif redundancy is a phenomenon which describes the many regulatory regions that contain a multitude of compensatory TF binding motifs [64]. Some regulatory mutations that remove TF motifs may not alter gene expression because compensatory binding elsewhere in the promoter region negates the effects of the altered TF motif, as illustrated in Figure 4. In addition, regulatory region redundancy may also be at play, whereby a gene is regulated by a number of different *cis*-regulatory regions. Melton, *et al*. [52] suggest that while this may increase the opportunity for regulatory somatic mutations that impact on the expression of a given gene to be acquired, it could also have the result of protecting against the effects of mutation. Therefore, while a mutation may still be pathogenic, TF and regulatory region redundancy ought to be considered, especially when designating functionality to a mutation in the absence of experimental validation.

### Identification of the genes associated with enhancer elements

Expression changes linked with TF alterations caused by somatic mutations can only be analysed in detail if the putative *cis*-regulatory region in which the mutation lies is able to be linked to an associated gene. This is a relatively simple process for promoter regions which are generally located upstream and in close proximity to the genes they regulate [15]. However, difficulties arise when enhancers are the target of analyses, as these regions engage with genes over vast distances [65] (Figure 1). Enhancer elements have been traditionally linked to genes by proximity, typically using the Genomic Regions Enrichment of Annotations Tool (GREAT) [66]. More recently however, studies have been increasingly adopting FANTOM5 – a data atlas containing mappings of enhancer-gene associations across the genome based on cap analysis of gene expression (CAGE) data correlations with putative target gene TSSs [67]. While the application of FANTOM5 data has allowed for more accurate designations of enhancer-gene associations, the atlas is not exhaustive for all enhancers genome-wide, nor for all cell-types available for study. It is due to these reasons perhaps, that there have not yet been large scale studies of somatic mutations from WGS data that occur specifically within enhancer regions of cancer genomes. In fact, to our knowledge, only a handful of somatic point mutations in enhancer regions have been experimentally linked to changes in gene expression, and potentially cancer development [52, 55, 60].

In cases where there is no FANTOM5 data available for a putative enhancer region, various experimental techniques are available to associate it with a genic region of the genome. These techniques involve chromosome conformation capture-based technologies such as Chromatin Interaction Analysis by Paired-End Tag (ChIA-PET) sequencing [68], Chromosome Capture Conformation (3C) [69], Circular Chromosome Conformation Capture (4C) [70], Carbon-Copy Chromosome Conformation Capture (5C) [71] and Hi-C [72]. These methods allow for the study of the spatial organization of genomic DNA by fixing chromatin interactions and sequencing ligation products to identify associations between distant parts of the genome [73]. In fact, 4C was used to identify the effects of the oncogenic *EVI1/GATA2* enhancer rearrangement in AML [49].

**Figure 5:**
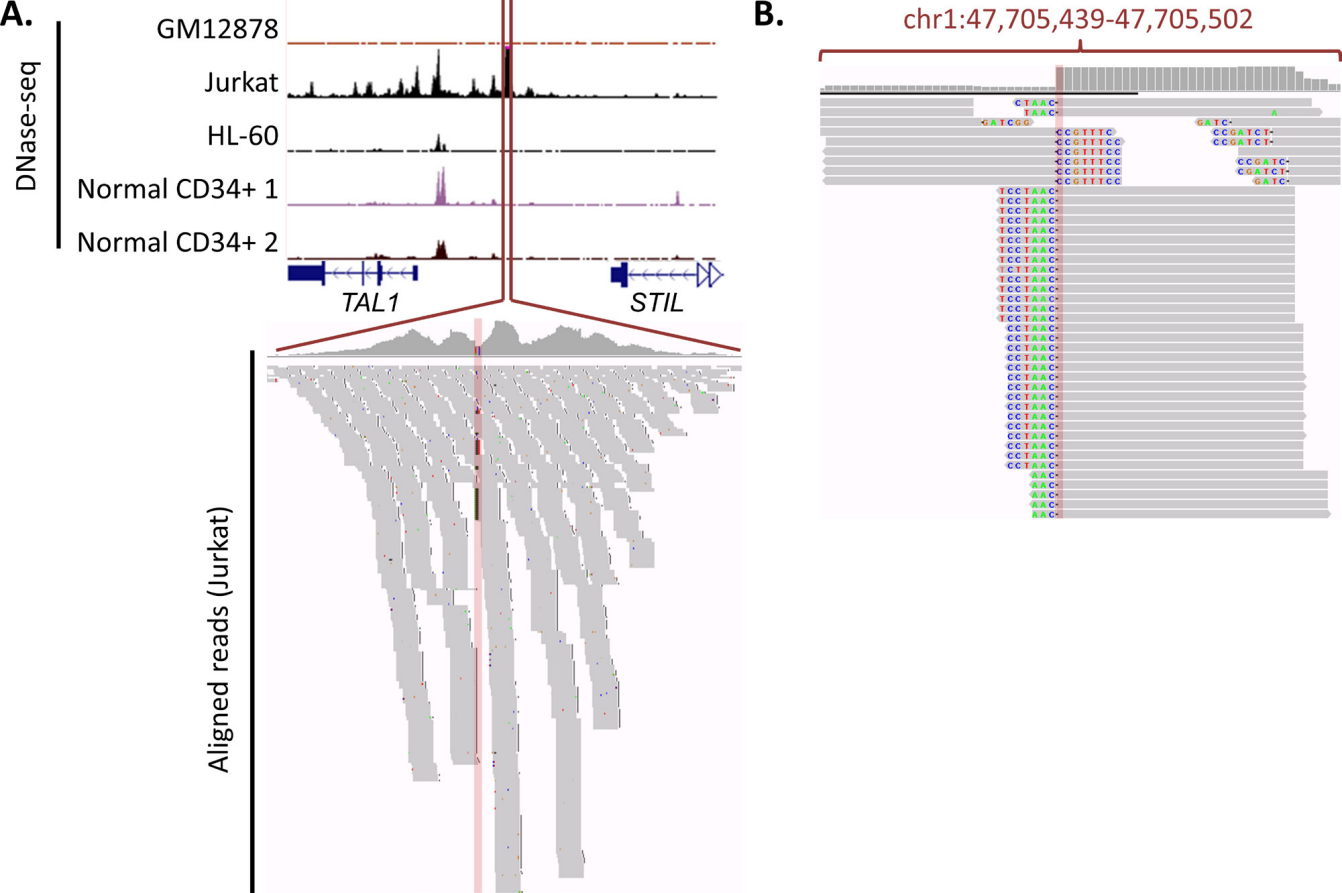
Gain of TAL1 enhancer mutation, along with DNase-seq data and sequencing reads **A.** DNase-seq data around *TAL1-STIL* locus. DNase-seq data (obtained from the ENCODE database [89]) is shown for a number of cell-types, indicating that the gain of an enhancer is unique to Jurkat cells. All tracks are fixed at 200 read coverage. The Jurkat aligned reads that correspond show a number of mismatches at the insertion site. **B.** Visualisation of soft-clipping reads. Soft-clipping DNase-seq reads from the Jurkat cell line (visualisation taken from the Integrative Genomics Viewer). Thorvaldsdóttir H, Robinson JT, Mesirov JP. Integrative Genomics Viewer (IGV): high-performance genomics data visualization and exploration. Brief Bioinform. 2013; 14: 178–192. Robinson JT, Thorvaldsdottir H, Winckler W, Guttman M, Lander ES, Getz G, Mesirov JP. Integrative genomics viewer. Nat Biotechnol. 2011; 29: 24–26. show a clear presence of reads corresponding with the insert. This demonstrates the way in which *cis*-regulatory variants can be identified from DNase-seq data alone.

Researchers may also need to use experimental techniques, such as chromatin conformation capture, ChIP-seq or DNase-seq, when they aim to determine sample-specific or somatically-created enhancer-gene associations. A potential approach that can be used to identify functional somatic mutations that create novel *cis*-regulatory regions is to make use of this sample-specific data to identify potential regulatory regions unique to specific cancer samples, and then seek to identify somatic variants within these regions. For example, Mansour *et al*. [46] used sample-specific data to identify the somatic *TAL1* enhancer alteration, as it did not fall into an enhancer region previously described in CD34 cells. As shown in Figure 5A, only by using sample-specific DNase-seq data from a cell line possessing the mutation (for example, Jurkat) could the DHS region created by the enhancer be identified. In fact, sequencing reads from DNase-seq can, in the absence of WGS, be used to identify somatic mutations uniquely present in DHS regions. Using the *TAL1* enhancer in Jurkat cells as an example, this concept is illustrated in Figure 5B, whereby clipped DNase-seq reads corresponding to the insertion mutation can be found within the enhancer.

### Increasing data availability in analyses

**Figure 6:**
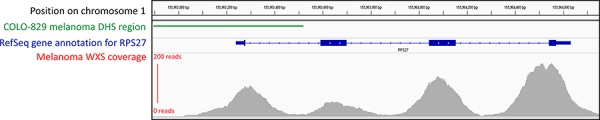
Read coverage for a sample gene using whole-exome sequencing (WXS) data, showing that sequencing can extend into intronic and promoter regions This figure depicts the read coverage of a WXS TCGA skin cutaneous melanoma sample (example used: TCGA-DA-A960) obtained from TCGA database. The region selected surrounds the *RPS27* gene, in which a recurrent 5′ UTR mutation was recently discovered in melanoma through the use of WXS data [90]. The approximate promoter (indicated by a DHS region) is shown using DNase-seq peak data from the COLO-829 malignant melanoma cell line, obtained from the ENCODE database [89]. Visualisation of all tracks is taken from the Integrative Genomics Viewer. Thorvaldsdóttir H, Robinson JT, Mesirov JP. Integrative Genomics Viewer (IGV): high-performance genomics data visualization and exploration. Brief Bioinform. 2013; 14: 178–192. Robinson JT, Thorvaldsdottir H, Winckler W, Guttman M, Lander ES, Getz G, Mesirov JP. Integrative genomics viewer. Nat Biotechnol. 2011; 29: 24–26.

Recent analyses have been performed on combined WGS data from many different cancer types (for example, 26 cancers [6], 14 cancers [51], 12 cancers [56] and 8 cancers [52]). However, no single cancer yet has enough individual samples with WGS to allow for the identification of non-coding mutations at low frequency in only a single cancer type [6]. In fact, many recent analyses have cited a need for more sequencing or matched expression data in order to draw firmer conclusions or identify mutations present at lower frequencies [6, 51, 52, 56]. It is worth noting that many sample sizes can be increased by the inclusion of whole-exome sequencing (WXS) data, which can often extend past the intended capture region [74] or span the intergenic space between adjacent genes and unintentionally overlap promoters (Figure 6). WXS data is therefore, a potential source of information that can be used to boost the statistical power of analyses of somatic promoter mutations while awaiting the WGS of further cancer samples. WXS data has already been used to some extent to establish the recurrence of certain promoter mutations [6, 51, 54], but to the best of our knowledge, there has been no large-scale application in non-coding genome research.

However, increases in data availability will inevitably also lead to increasing complexity. As the pool of potential candidate driver mutations identified from cancer WGS datasets increases, better bioinformatic screening will be required to determine those mutations which ought to be segregated for targeted experimental analysis. This highlights the importance of accounting for mutational heterogeneity (using such models as MutSigCV [75]) and false positive mutation calls arising due to mapping errors [52]. Without such analyses, candidate recurrent somatic mutations may be spuriously identified if they simply fall into mutagenic hotspots or regions for which the mapping of reads is difficult.

Further, if recurrence continues to be adopted as a key indicator of potential driver mutations, cases must be considered where a given gene is regulated by a number of *cis*-regulatory regions located in different areas of the genome. Each region may potentially harbour only a small number of somatic mutations that wouldn't be identified by simple statistical analyses. However, the mutations may be arranged in such a way that the associated gene will be influenced by a statistically significant number of *cis*-regulatory mutations across samples. Alternatively, it may also be possible for a large genomic window to harbour a complex pattern of mutations within a single sample, but not be detected due to the decreased statistical power of analyses of such large regions [51]. Therefore, analyses performed on large datasets may potentially miss genes with such mutational patterns, or a significant number of mutations in their *cis*-regulatory regions, if the biological function and interaction of these regions is not considered.

### Strategies to improve targeting of *cis*-regulatory regions and mutations

Additional criteria may help researchers to determine the best candidates for experimental analyses. For example, with many oncogenes and tumour suppressor genes regulated by strong enhancers, it is worth noting that strong enhancers are more sensitive to alterations that decrease the activity of their transcriptional regulators [48]. With this in mind, perhaps research focus could be particularly drawn to super-enhancers and other strong enhancers in the cancer genome to allow for more effective targeting of potential candidate mutations [48]. Strong enhancers such as super-enhancers can be identified by the presence of particularly high levels of binding by Mediator (Med1) [76].

For further improved targeting, analysis of mutations falling into ultra-sensitive regions [77] may elucidate the most deleterious mutations. Additionally, on the assumption that highly conserved bases will be the most sensitive to mutations, human population-variation data could be used to better target specific bases, rather than cross-species conservation [77], as many regions that are conserved in a variety of mammals are not active in humans [78].

Finally, it is worth considering the interaction between somatic mutations in regulatory regions of genes whose protein-coding exons are often mutated in cancer. As previously discussed, co-occurrence of coding and regulatory mutations [51, 54] may lead to previously unknown cancer mechanisms. It is possible that exonic and promoter mutations may form complementary mechanisms by which expression is altered in cancer. Mathelier *et al*. [53] has suggested this with reference to *ID3*, a gene with TF binding site mutations and recurrent coding mutations in Burkitt lymphoma. Hence, promoter or enhancer mutations in the regulatory regions of genes that are often mutated in a given cancer type may provide a good filtering mechanism for candidate driver somatic mutations.

## AVAILABLE WEB SERVERS

For researchers less familiar with, or with less access to, genome-wide cancer datasets, a number of web servers exist [55, 79–83] that provide annotations of putative *cis*-regulatory variations (see Table 2). These tools can allow researchers to identify the mutations in a dataset that are the most likely to be functional. A number of these tools provide scorings of each mutation or variant analysed, allowing researchers to better prioritise candidate mutations. Of note, the FunSeq2 server additionally incorporates network analysis to connect non-coding variants into protein-protein, regulatory and phosphorylation networks, providing a measurement that indicates the likelihood of a variant being deleterious [79]. Additionally, while the OncoCis web server [55] does not score mutations, it is specifically designed for the annotation of somatic cancer mutations in *cis*-regulatory regions. It utilises cell-type-specific histone marks to identify mutations in putative promoters and enhancers. The need to account for tissue specificity when identifying and characterizing *cis*-regulatory regions is becoming increasingly evident [53, 55, 77, 84] as mutations may be deleterious only in certain tissues [77] and many *cis*-regulatory regions are not ubiquitously involved in gene expression across tissue types [67]. This tool thus helps to identify the somatic mutations in a dataset that are most likely to be functional in a relevant cell-type.

**Table 2:** Tools available for analysis of the functional role of non-coding variants and somatic mutations

Tools	Description*	Web link
CADD [83]	Combined Annotation-Dependent Depletion (CADD) integrates a number of annotations to provide a C score which represents the likelihood of deleteriousness of a single nucleotide variant or small indel. CADD can be used for both somatic and germline variants, in coding and non-coding regions of the genome.	http://cadd.gs.washington.edu/
FATHMM-MKL [82]	FATHMM-MKL is a machine learning approach that uses a variety of predictive measures such as conservation, histone modification, transcription factor binding and GC content. It can be applied in the analysis of both somatic and germline variants in coding and non-coding regions of the genome.	http://fathmm.biocompute.org.uk/fathmmMKL.htm
FunSeq2 [79]	FunSeq2 is an analysis pipeline which provides a weighted scoring system based on conservation, transcription factor binding gain- or loss-of-function events, recurrence, enhancer-gene associations and network centrality. FunSeq2 has application for both somatic and germline non-coding variants.	http://funseq2.gersteinlab.org/
GWAVA [81]	Genome-Wide Annotation of Variants (GWAVA) is a tool which prioritises analysis of non-coding variants, and is designed to be applied for both germline and somatic variants. It uses a variety of both genomic and epigenomic annotation methods to provide a GWAVA score allowing identification of likely functional variants.	https://www.sanger.ac.uk/sanger/StatGen_Gwava
OncoCis [55]	OncoCis is a webserver which allows researchers to identify *cis*-regulatory somatic mutations by using conservation, transcription factor binding and cell-type-specific genome and epigenome datasets. The tool is designed for use with non-coding somatic mutations from cancer datasets and can incorporate matched expression data.	https://powcs.med.unsw.edu.au/OncoCis/
RegulomeDB [80]	RegulomeDB scores regulatory variants to prioritise those variants that have functional consequences. The tool applies such data as eQTL, ChIP-seq, DNase-seq and TF motifs. It is targeted at the annotation of germline variants but can be used in analysis of somatic mutations. It is designed for use in annotating non-coding variants.	http://regulomedb.org/

* See reference or web link provided for a fuller description of each of the tools listed.

## CONCLUDING REMARKS

No *cis*-regulatory somatic mutations have yet been identified in recent studies that have such strong associations with altered gene expression and cancer development as the regulatory mutations impacting *TERT* and *TAL1*. Perhaps this is because somatic *cis*-regulatory mutations play only a minor role in cancer development, with the *TERT* promoter mutations being exceptional in their recurrence both within and across cancer types. However, with increasing evidence that germline variants play a driver role in cancer risk, this conclusion seems unlikely. It is our opinion that it is more probable that other *cis*-regulatory mutations exist at lower prevalence, or in only one cancer type, and simply have not yet been identified. Thousands of WGS samples will likely be required to accurately identify driver mutations that are present at low frequencies among the background of passenger mutations in the cancer genome [85]. For this reason, further analysis will be required in order to uncover the true role of *cis*-regulatory somatic mutations in cancer development.

This research is vital, as cancer driver mutations in *cis*-regulatory regions may potentially serve as biomarkers or drug targets [37, 86]. For example, drugs targeting enhancer regions can be used in therapy in cases where cancer is driven by aberrant enhancer regulation [49]. In fact, tumour-specific super-enhancers are preferentially targeted by drugs that act on components of the transcriptional complex [46], and this area will potentially produce breakthrough results. Other targets include the TFs that are recruited to the mutated *cis*-regulatory regions of important cancer driver genes, which may provide a means of halting cancer progression [87]. Thus, research into *cis*-regulatory somatic cancer mutations may produce potentially fruitful therapeutic targets. It is our hope that the recommendations made in this review for future research direction will help in this endeavour.
